# Publisher Correction: In-plane and out-of-plane excitonic coupling in 2D molecular crystals

**DOI:** 10.1038/s41467-023-38971-y

**Published:** 2023-05-31

**Authors:** Dogyeong Kim, Sol Lee, Jiwon Park, Jinho Lee, Hee Cheul Choi, Kwanpyo Kim, Sunmin Ryu

**Affiliations:** 1 Department of Chemistry, Pohang University of Science and Technology (POSTECH), Pohang, Gyeongbuk 37673 Korea; 2 Department of Physics, Yonsei University, Seoul, 03722 Korea; 3 Center for Nanomedicine, Institute for Basic Science (IBS), Seoul, 03722 Korea

**Keywords:** Fluorescence spectroscopy, Excited states, Chemical physics

Correction to: *Nature Communications* 10.1038/s41467-023-38438-0, published online 12 May 2023

The original version of this Article contained an error in Fig. 5, in which the two magenta arrows in panels b and c were displaced and their heads were oversized.

This has been corrected in both the PDF and HTML versions of the Article.

