# Supplementary material for: In-plane and out-of-plane excitonic coupling in 2D molecular crystals
Source: Nat Commun. 2023 May 12;14:2736. doi: 10.1038/s41467-023-38438-0 (PMC10182054; doi:10.1038/s41467-023-38438-0)
Supplement: Supplementary file 1 — Supplementary Information [file 41467_2023_38438_MOESM1_ESM.pdf]

Supplementary Information for

In-Plane and Out-of-Plane Excitonic Coupling in 2D Molecular Crystals

Dogyeong Kim, Sol Lee, Jiwon Park, Jinho Lee, Hee Cheul Choi, Kwanpyo Kim and Sunmin Ryu*

Correspondence to: sunryu@postech.ac.kr

This PDF file includes:

Supplementary Table (1)
Supplementary Figures (1 ~ 12)
Supplementary Note (1)
Supplementary References

Supplementary Table

Supplementary Table 1. Lattice constants of 2D PTCDA crystals on 2D hBN and graphene. The data for bulk crystals by Möbus et al.[1] are also compared. All lengths are in nm.

	**$1L_P$/hBN**	**$3L_P$/hBN**	**$1L_P$/Gr**	**$3L_P$/Gr**	**Bulk**	
**Phase**	-	**α**	-	**α**	**α**	**β**
Space group	-	-	-	-	$P2_1/c$	$P2_1/c$
a					0.374	0.378
b					1.196	1.930
c					1.734	1.077
β (deg)					98.8	83.6
m	1.246	1.186	1.267 ± 0.001	1.238 ± 0.005	1.196	1.930
n	1.991	1.981	1.961± 0.001	1.968 ± 0.003	1.991	1.245
∠ *mn* (deg)	89.5	89.4	89.4 ± 0.2	90.38 ± 0.11	90	90
d	0.31 ± 0.08	0.37 ± 0.11	-	0.37 ± 0.26	0.322	0.325

Supplementary Figures

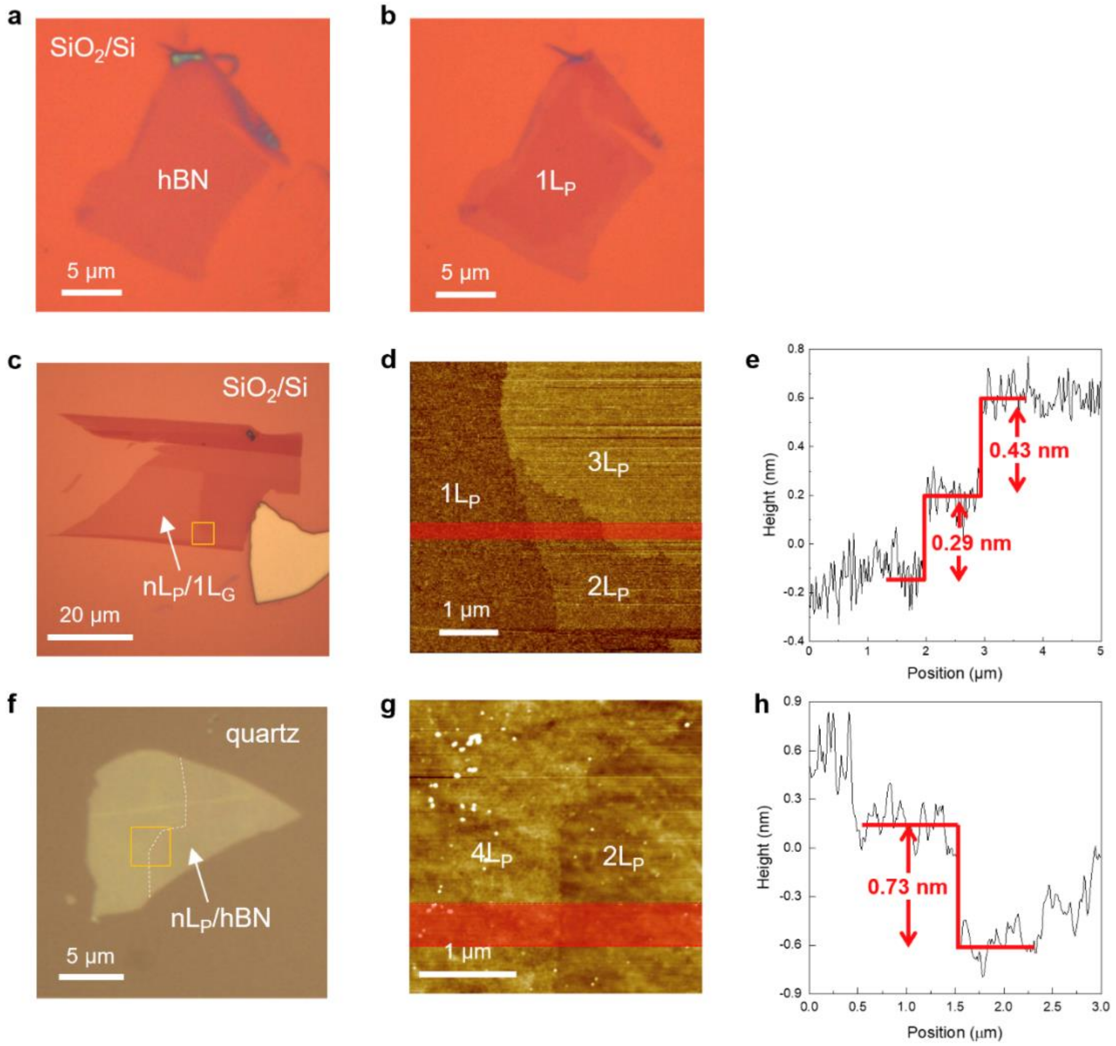

Supplementary Figure 1. Optical and topographic images of 2D PTCDA crystals on various substrates. (a & b) Optical micrographs of a few-layer hBN crystal mechanically exfoliated on a SiO_2/Si substrate obtained before (a) and after (b) growth of $1L_P$. (c ~ e) Optical micrograph (c), AFM height image (d), and height profile (e) of $1L_P$ ~ $3L_P$ grown on $1L_G/SiO_2$/Si. The AFM image was obtained from the yellow square in (c), and the height profile was averaged over the red box in (d). (f ~ h) Similar data of $2L_P$ and $4L_P$ grown on hBN/quartz as (c ~ e).

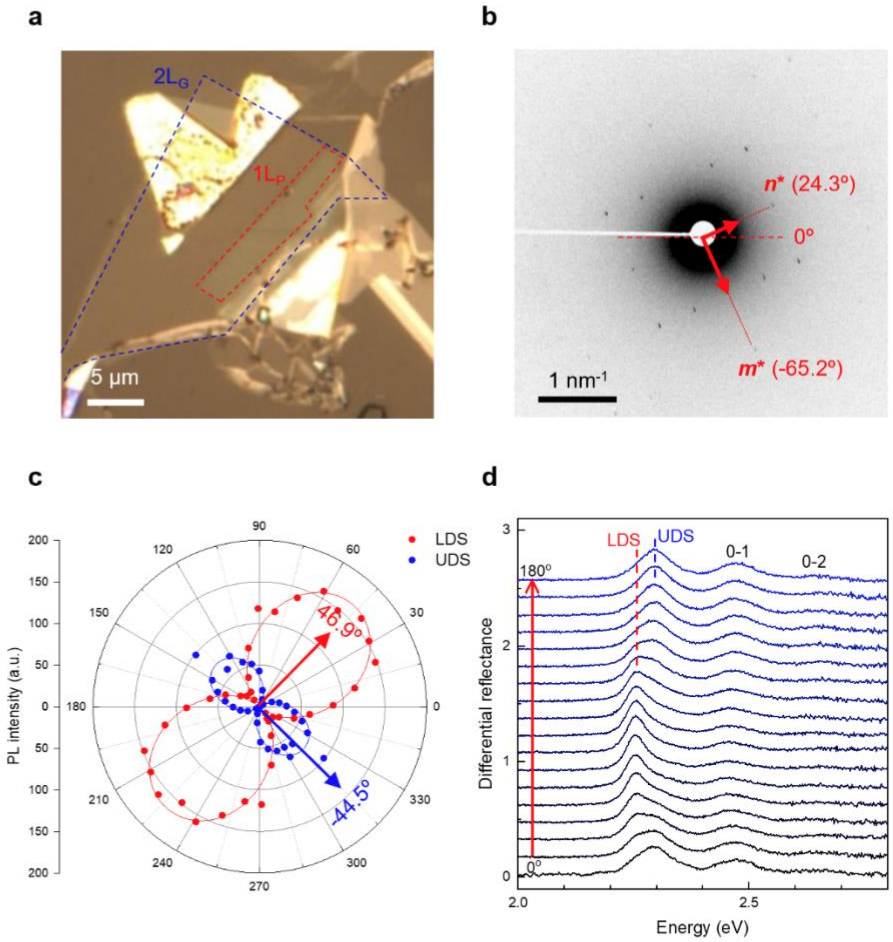

Supplementary Figure 2. Correlative determination of crystallographic and optical axes of 2D PTCDA crystals. (a) Optical micrograph of $2L_G/1L_P$/hBN. The edges of the constituent layers are marked with blue ($2L_G$) and red ($1L_P$) lines, whereas those of the underlying hBN are located outside the image. (b) Same SAED image as Fig. 1c, obtained from $1L_P$ area in (a). (c) Parallel-configuration PL intensity of LDS and UDS peaks from $1L_P$ in (a). The data were fitted with the square of cosine. The angle between μ_{LDS} and $\boldsymbol{n}$ shown in Fig. 2e was obtained by comparing (b) and (c): the real-space orientations of μ_{LDS} and $\boldsymbol{n}$ could be determined with respect to linear edges that are present within samples. (d) Parallel-configuration differential reflectance (DR) spectra of $1L_P$/hBN/quartz. Because DR is proportional to absorptance (**A**), the angle ($\theta_{I/II}$) between two molecular bases shown in Fig. 2e could be determined as follows. The absorptance ratio between UDS and LDS ($r = \mathbf{A}_{UDS}/\mathbf{A}_{LDS}$) is identical to $|\mu_{UDS}|^2/|\mu_{LDS}|^2$ and can be given as a function of $\theta_{I/II}$ using trigonometric relations. One can further show that $\theta_{I/II} = \cos^{-1}[(1 - r)/(1 + r)]$.

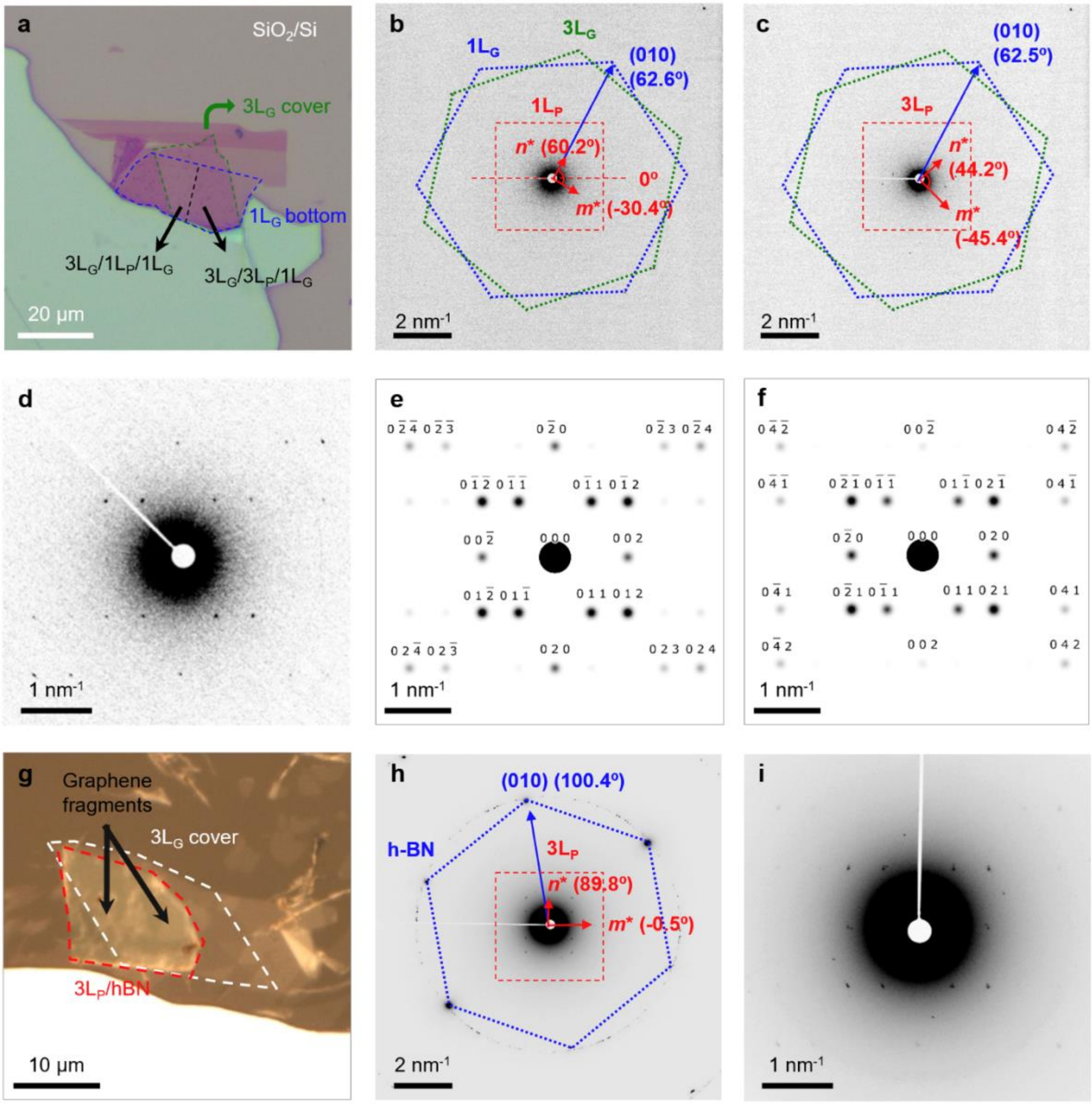

Supplementary Figure 3. Polytype determination of multilayer 2D PTCDA crystals. (a ~ d) Optical micrograph (a) and SAED patterns (b ~ d) of $3L_G/1L_P/1L_G$ (b) and $3L_G/3L_P/1L_G$ (c, d). The hexagonal patterns from graphene are marked with blue ($1L_G$) and green ($3L_G$) dotted lines in (b & c), whereas the signals from PTCDA are black dots in (d). (e & f) Simulated SAED patterns of α- and β-PTCDA crystals shown in (e) and (f), respectively. (g ~ i) Optical micrograph (g) and SAED patterns (h & i) of $3L_G/3L_P$/hBN. The data in (d) and (i) indicate that 2D PTCDA crystals are closer to α-polymorph in (e).

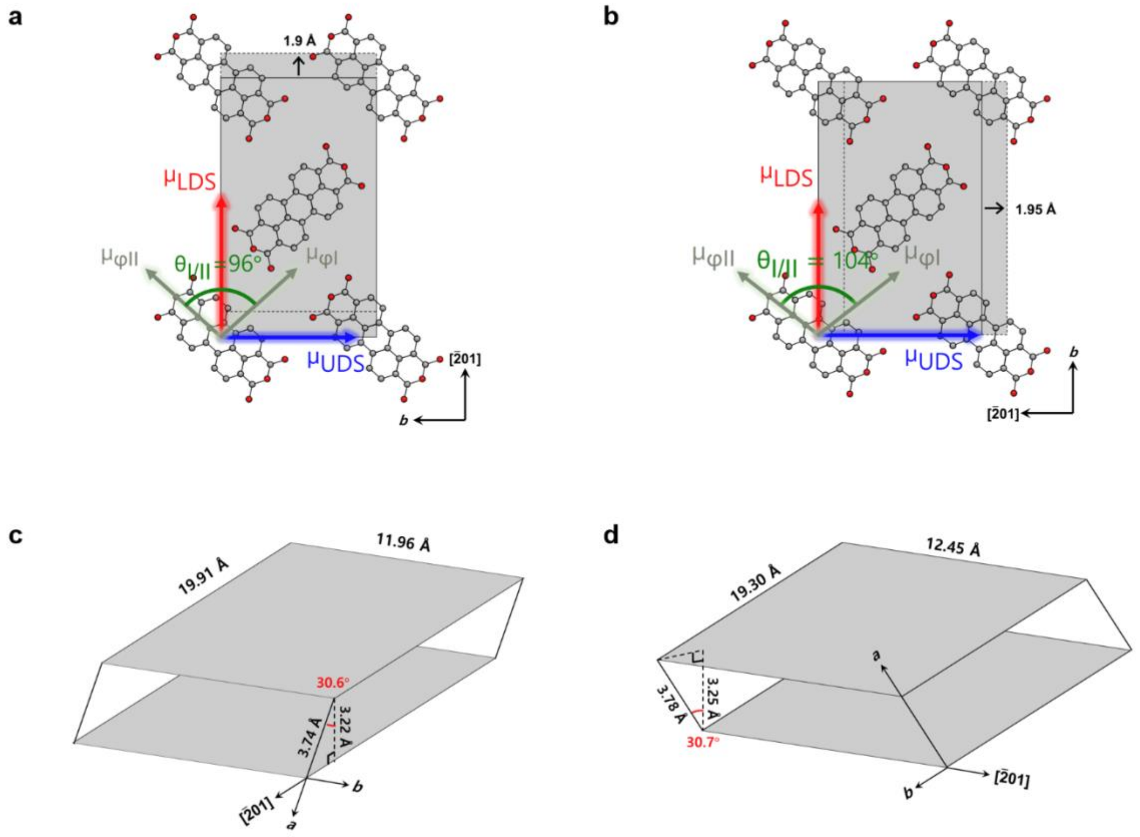

Supplementary Figure 4. Structure of bulk PTCDA crystals. (a & b) Top view over (102) plane spanned by ***b*** and $[\bar{2}01]$ of α- (a) and β-phase (b) crystals. The solid and dotted rectangles represent 2D unit cells in two neighboring layers. The unit cells are shifted against each other by 1.90 Å along the long axis for α-phase and 1.95 Å along the short axis for β-phase. (c & d) Schematic unit cells of α- (c) and β-phase (d) crystals with lattice parameters.[1]

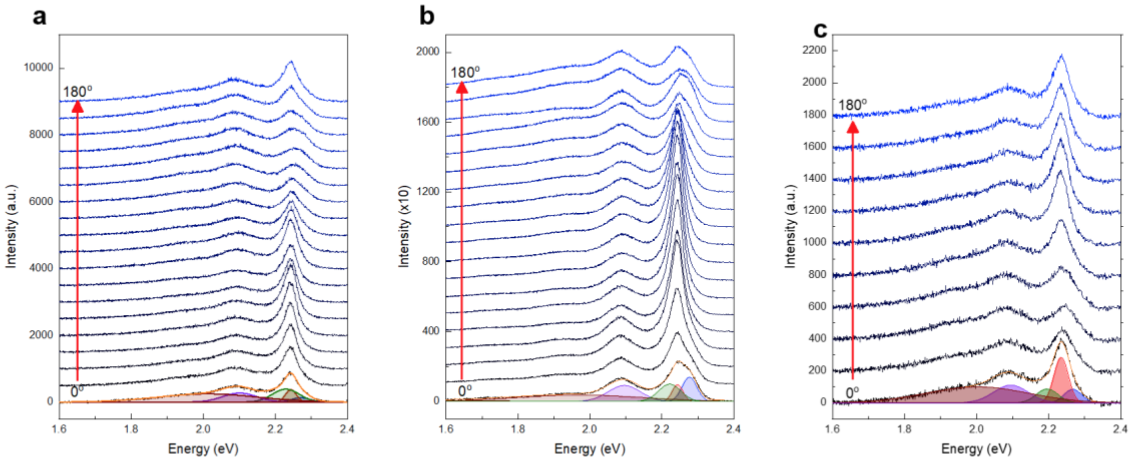

d

Energy (eV)	UDS	LDS	*	0-1	0-2
Set a	2.271	2.243	2.229	2.101	2.012
Set b	2.277	2.244	2.222	2.096	1.973
Set c	2.268	2.235	2.196	2.096	2.002
Average	2.272	2.241	2.216	2.098	1.996

Supplementary Figure 5. Complete polarized PL spectra of 1L$_P$/hBN samples. (a ~ c) All data were obtained in a parallel configuration. The data shown in Fig. 2b belong to (a). Each set was globally fitted with five Gaussian functions (shaded in various colors). (d) Energies of the five components. Whereas the peak denoted by an asterisk (in green shades) improved the global fitting, its origin is unclear.

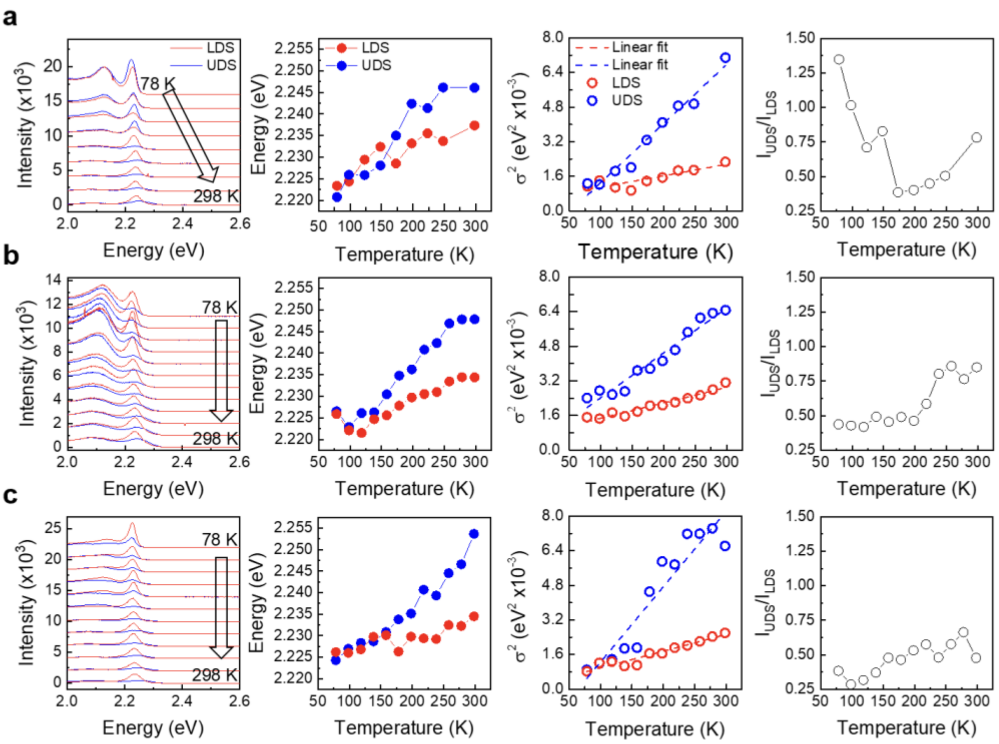

Supplementary Figure 6. Additional data sets of temperature-dependent PL spectra. (a ~ c) 2L$_P$/hBN (a) and 1L$_P$/hBN samples (b & c). The red and blue spectra in the first column are LDS- and UDS-polarized, respectively. Red (blue) symbols in the second and third columns represent the peak energy and σ^2 of the LDS (UDS) transition, respectively. The last column shows the intensity ratios of UDS and LDS peaks.

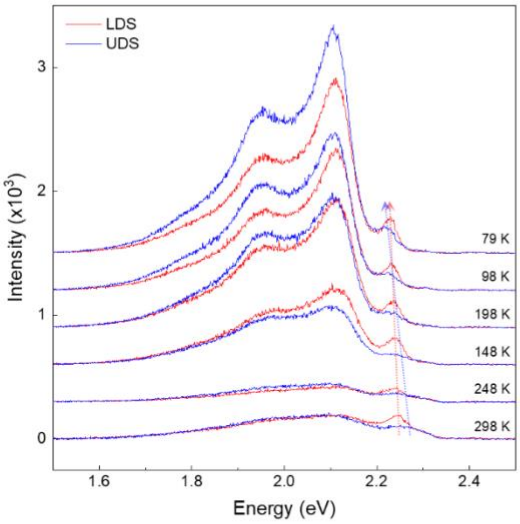

Supplementary Figure 7. Complete polarized PL spectra of 1L$_P$/hBN shown in Fig. 3. Dotted guidelines indicate that LDS and UDS peaks exhibit energy inversion with decreasing temperature. Note that the low-energy emissions for 0-1 and 0-2 vibronic transitions at 298 K are stronger than other 1L$_P$ samples shown in Figs. 1f, 2b and S5. The additional contributions are made from CT states that reside in a small fraction of multilayer patches of this particular sample. This can be seen by comparing the low-temperature PL spectra, dominated by the CT states, of this sample with those from others (e.g., Supplementary Figure 6c).

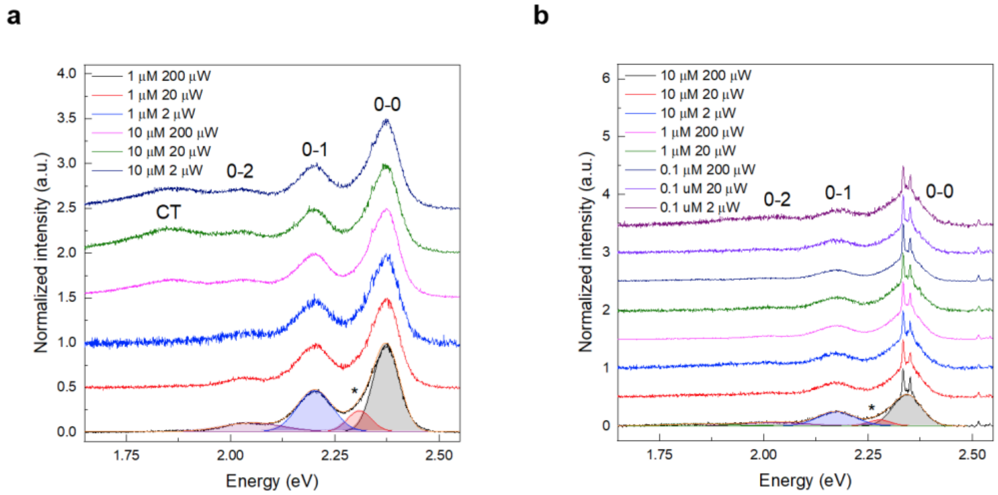

Supplementary Figure 8. Unpolarized PL spectra of monomeric PTCDA. (a & b) PTCDA solutions in chloroform (a) and toluene (b) for various concentrations and excitation powers. Data were fitted with four Gaussian functions (in color shades). The broad CT band at 1.85 eV in (a) suggests the formation of PTCDA aggregates at a high concentration. Whereas the peak denoted by an asterisk at ~2.3 eV improved the fitting, its origin is unclear. Two sharp peaks on top of 0-0 emission in (b) are Raman signals of the solvent.

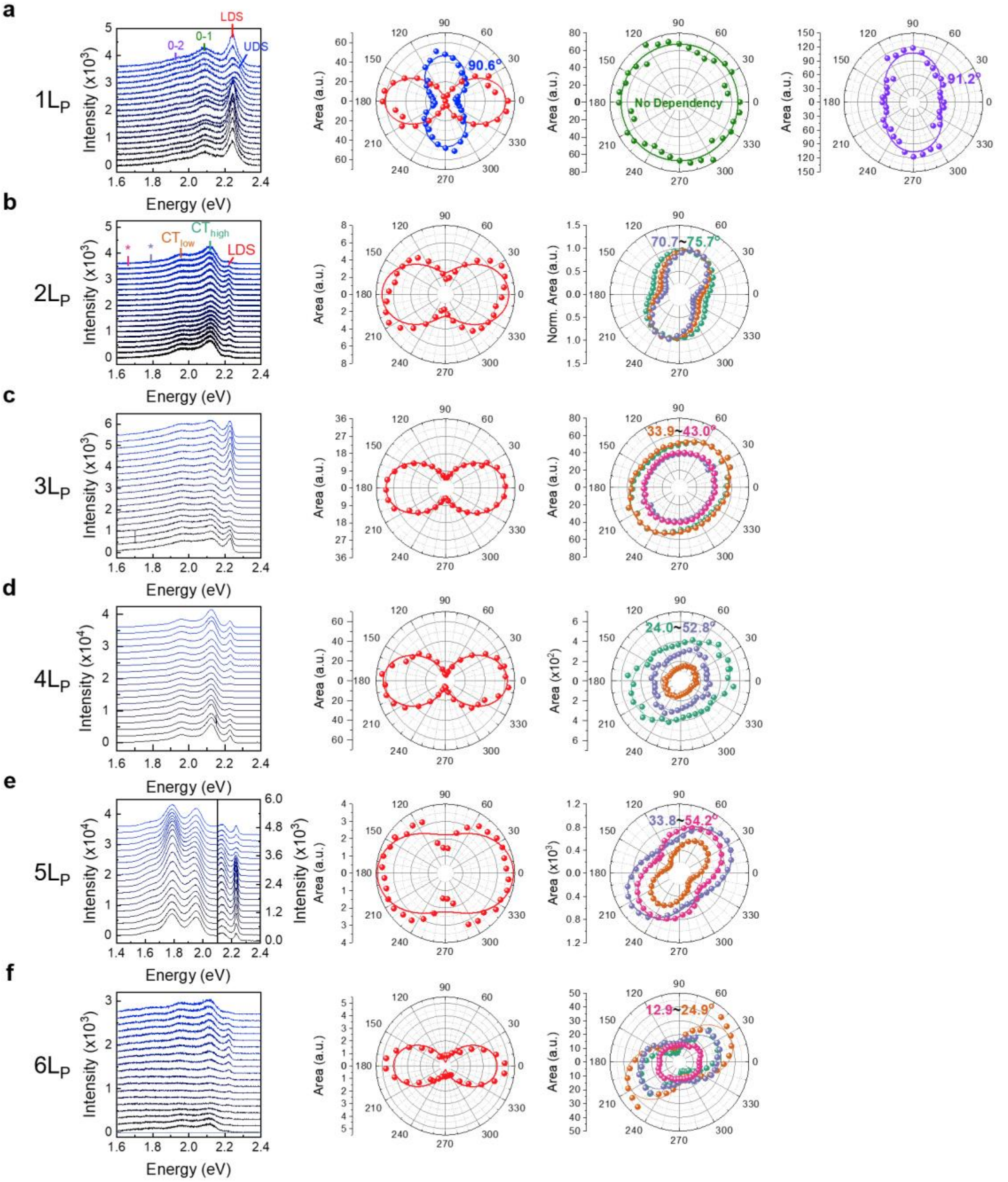

Supplementary Figure 9. Polarized PL spectra of nL_P/hBN. (a) PL spectra of $1L_P$ (first column), and their polar intensity graphs of LDS & UDS (second column), 0-1 (third column) and 0-2 (fourth column) peaks. (b ~ f) PL spectra of multilayer nL_P (first column), and their polar intensity graphs of LDS (second column) and CT peaks (third column). Two broad unresolved peaks (marked with asterisks) correspond to vibrational progressions of CT excitons.[2] Polar graphs were colored as their peak labels. Whereas the multilayer data (b ~ f) were obtained at 77 K, the measurements for $1L_P$ (a) were performed at 298 K for the best separation of the two DS peaks. The solid lines in polar graphs are a modified Malus fit to the data in the form of "$\cos^2(\theta - \theta_o)$ + constant", where θ_o (also noted next to each ellipse) represents the orientation of the major axis of ellipses.

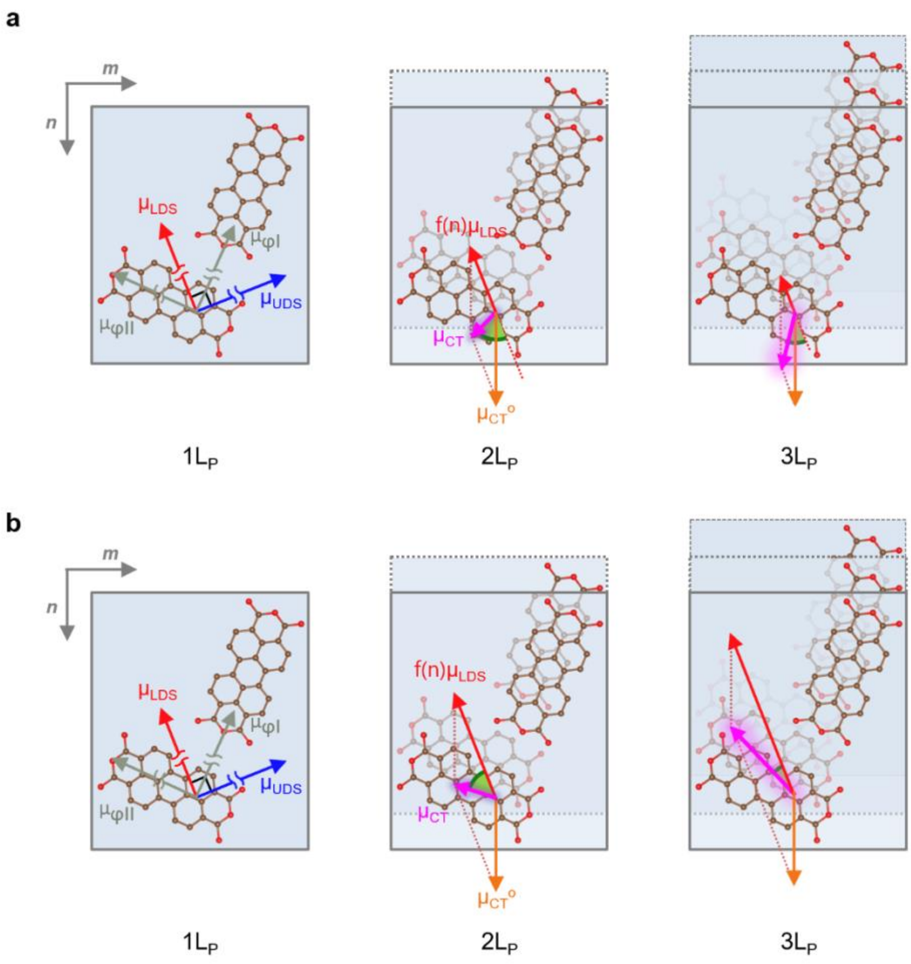

Supplementary Figure 10. Thickness-dependent reorientation of μ_{CT}. (a) When the contribution of FE° decreases with increasing thickness (n): For $1L_P$ (left), μ_{LDS} and μ_{UDS} are formed orthogonal. For $2L_P$ (middle), $\mu_{CT}{}^{o}$ aligned along ***n*** axis is modified by μ_{LDS} and mixed into μ_{CT} (magenta arrow). For $3L_P$ (right), μ_{CT} is reoriented further towards ***n*** axis because of the decreased Frenkel contribution. (b) When the contribution of FE° rises with increasing thickness (n): μ_{CT} is eventually reoriented towards μ_{LDS}. Whereas the magnitude of $\mu_{CT}{}^{o}$ is maintained constant, the contribution of FE° is varied by the thickness-dependent mixing factor, f(n), in (a) and (b). See Supplementary Note 1 for details.

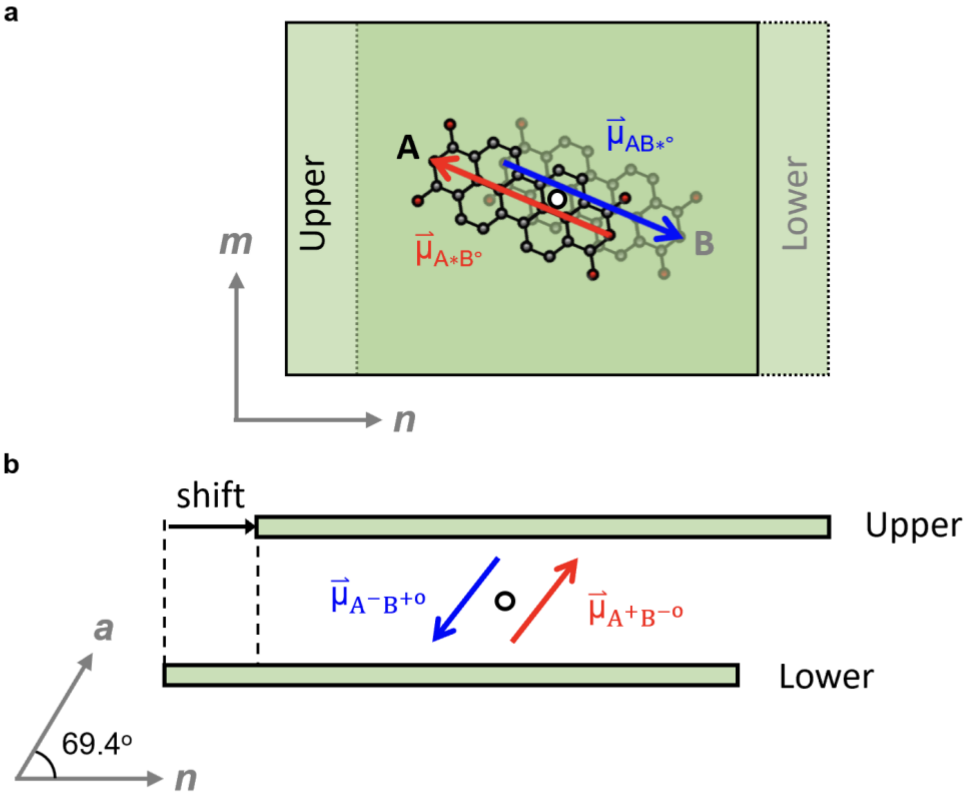

Supplementary Figure 11. Symmetry of transition dipole moments of a dimer system representing 2Lp. (a) Two equivalent $\vec{\mu}_{FE^{\circ}}$ with excitation located in the upper ($\vec{\mu}_{A^*B^{\circ}}$) and lower ($\vec{\mu}_{AB^{*\circ}}$) molecules. Because of the inversion center (black circle), anti-parallel phase convention was adopted. (b) Transition dipole moments for A-to-B electron ($\vec{\mu}_{A^+B^{-\circ}}$) and hole ($\vec{\mu}_{A^-B^{+\circ}}$) transfer are also anti-parallel to each other. For details, see Supporting Information (Supplementary Note 1). Theory predicted that $\vec{\mu}_{A^+B^{-\circ}}$ and (102) planes form 26.1°,[3] which is much smaller than 69.4° between (102) plane and the stacking axis of α-phase denoted as ***a***.

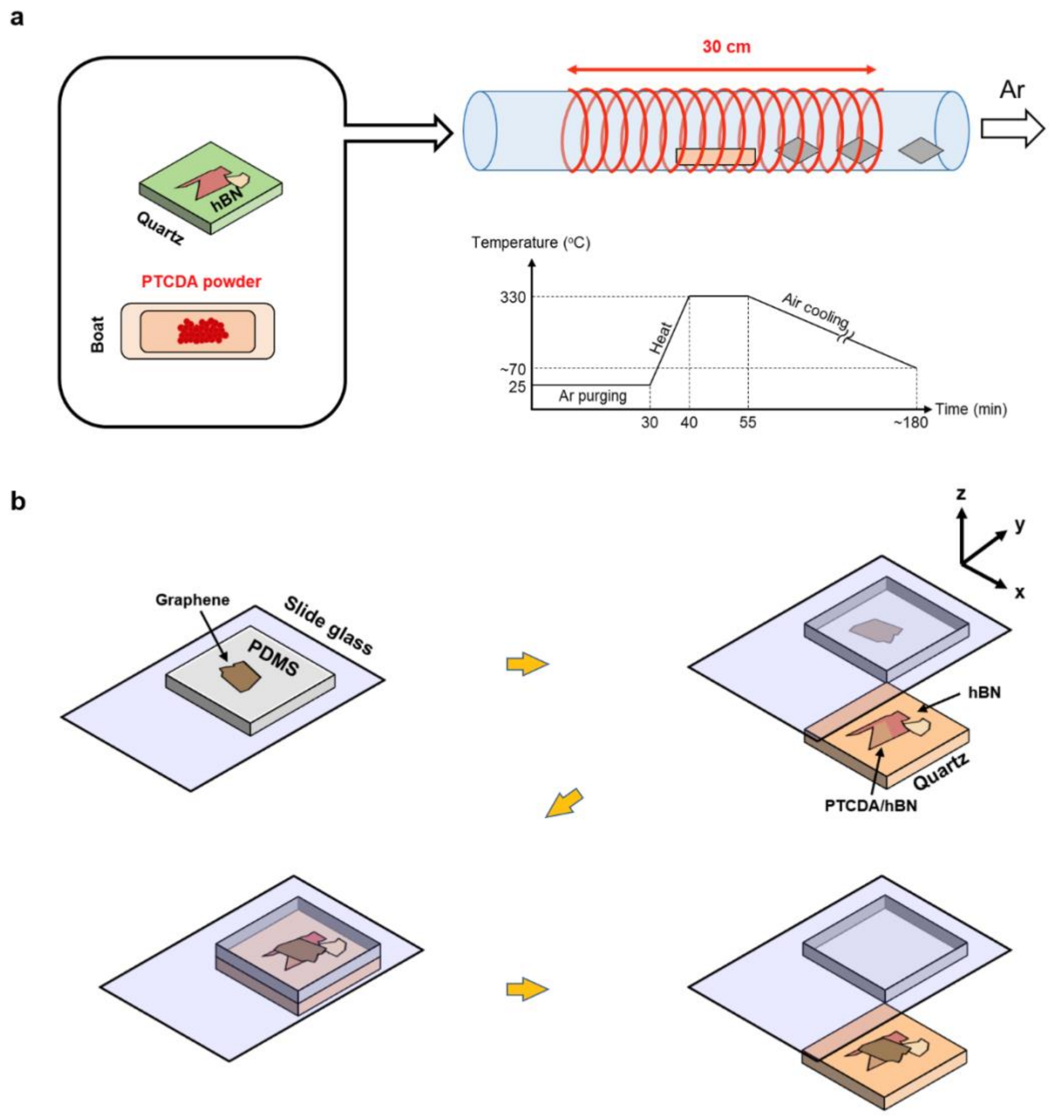

Supplementary Figure 12. Experimental scheme for the fabrication of sandwiched nL_P samples. (a) Physical vapor deposition (PVD) of PTCDA on exfoliated hBN (or graphene) in a flow-type tube furnace. (b) Dry transfer of graphene layers on top of nL_P/hBN samples. See Methods for the details.

Supplementary Note

Supplementary Note 1. Frenkel-CT mixing in 2D PTCDA

We showed that the transition dipole moments of the CT states (μ_{CT}) are reoriented within (102) plane as the thickness of 2D PTCDA varies (Figs. 5 and S9). Accepting the validity of the 1D chain model by Hoffmann et al.,[4] μ_{CT} would be virtually fixed because CT would occur along the 1D stack of PTCDA, which contradicts the observed reorientation. Instead, we propose that the CT state is mixed with Frenkel excitons (FE),[5] more specifically LDS, as depicted in Fig. 5e. This scenario is corroborated by two facts. First, the DS energy of 31 meV for $1L_P$ indicates that the Coulombic interaction between neighboring I & II bases is substantial. Thus, the inter-stack coupling cannot be neglected in describing the CT states of multilayers. Second, the highest CT state is very close (within ~100 meV) to the LDS of $1L_P$ (Supplementary Figure 9), suggesting a significant mixing between both states. We note that a similar Frenkel-CT mixing was observed for tetracene crystals with an energy difference of ~300 meV.[6, 7]

Below, we present a simple model approach for FE-CT mixing in 2D PTCDA crystals. For $2L_P$, each 1D stack contains two π-stacked PTCDA molecules, A & B, as depicted in Fig. 5f. The coupling between two stacks of basis I and II leads to the separation of LDS and UDS (Fig. 2a). The CT states ($|A^+B^-\rangle$ or $|A^-B^+\rangle$) affected by unperturbed Frenkel states ($|A^*B\rangle$ or $|AB^*\rangle$) can be approximated using the first-order perturbation theory[8] as follows:

$$|A^+B^-\rangle = |A^+B^-\rangle^\circ - \lambda|A^*B\rangle - \kappa|AB^*\rangle \tag{S1}$$

$$|A^-B^+\rangle = |A^-B^+\rangle^\circ - \lambda|AB^*\rangle - \kappa|A^*B\rangle \tag{S2}$$

, where $|A^+B^-\rangle^\circ$ and $|A^-B^+\rangle^\circ$ are unperturbed CT states. Two coefficients, λ and κ, can be related to the electron and hole transfer integrals, respectively.[9] As shown in Fig. 5e, FE-CT mixing will affect LDS more than UDS because of their differing energetics. For simplicity, we assume that the mixing is solely due to LDS.

Because of its centrosymmetry, the CT state of the dimer, $\varphi_{CT}^{\pm}$, should be linear combinations of $|A^+B^-\rangle$ or $|A^-B^+\rangle$:

$$\begin{aligned}\varphi_{CT}^{\pm} &= \frac{1}{\sqrt{2}}(|A^+B^-\rangle \pm |A^-B^+\rangle) \\ &= \frac{1}{\sqrt{2}}\left(|A^+B^-\rangle^\circ \pm |A^-B^+\rangle^\circ\right) \\ &\quad - \frac{1}{\sqrt{2}}(\lambda \pm \kappa)(|A^*B\rangle \pm |AB^*\rangle)\end{aligned} \tag{S3}$$

The symmetric state (φ_{CT}^{+}) is dipole-forbidden because the two constituent transition dipole moments cancel each other ($\vec{\mu}_{A^+B^{-o}} = -\vec{\mu}_{A^-B^{+o}}$ and $\vec{\mu}_{A^*B^o} = -\vec{\mu}_{AB^{*o}}$). However, the difference between transition dipole moments of the antisymmetric state (φ_{CT}^{-}) is non-zero because of the centrosymmetry ($\vec{\mu}_{A^*B} = -\vec{\mu}_{AB^*}$). Then, the transition dipole moment of φ_{CT}^{-} can be given as follows:

$$\vec{\mu}_{CT}^{-} = \left\langle AB \middle| \vec{d} \middle| \frac{1}{\sqrt{2}}(|A^+B^-\rangle - |A^-B^+\rangle) \right\rangle \quad \text{(S4)}$$

$$\begin{aligned} &\vec{\mu}_{CT}^{-} \\ &= \left\langle AB \middle| \vec{d} \middle| \frac{1}{\sqrt{2}}\left(|A^+B^-\rangle^\circ - |A^-B^+\rangle^\circ\right) - \frac{1}{\sqrt{2}}(\lambda - \kappa)(|A^*B\rangle - |AB^*\rangle) \right\rangle \\ &= \vec{\mu}_{CT^\circ}^{-} - (\lambda - \kappa)\frac{2}{\sqrt{2}}\vec{\mu}_{FE^\circ} \end{aligned} \quad \text{(S5)}$$

, where $|AB\rangle$ is the ground state of the dimer and $\vec{d}$ is the dipole moment operator. $\vec{\mu}_{FE^\circ}$ is the transition dipole moment of the unperturbed FE state for a monomer and satisfies the following relation:

$$\vec{\mu}_{FE^\circ} = \langle A|\vec{d}|A^*\rangle = \langle AB|\vec{d}|A^*B\rangle = -\langle AB|\vec{d}|AB^*\rangle \quad \text{(S6)}$$

Also, note the following equality for $\vec{\mu}_{CT^\circ}^{-}$:

$$\begin{aligned} \vec{\mu}_{CT^\circ}^{-} &= \left\langle AB \middle| \vec{d} \middle| \frac{1}{\sqrt{2}}\left(|A^+B^-\rangle^\circ - |A^-B^+\rangle^\circ\right) \right\rangle = \frac{2}{\sqrt{2}}\langle AB|\vec{d}|A^+B^{-\circ}\rangle \\ &\equiv \frac{2}{\sqrt{2}}\vec{\mu}_{A^+B^{-o}} \end{aligned} \quad \text{(S7)}$$

Equation S5 above indicates that the polarization dependence of the CT state will be governed by the degree of the FE-CT mixing. Because the quantitative analysis of the mixing goes beyond the scope of this work, we will explore qualitatively what can be expected for two opposite cases where the degree of mixing decreases or increases with increasing thickness. The former case may appear reasonable when the above description is expanded to an n-mer for nLP. The unperturbed FE states of the n-mer consist of n linear combinations of n monomer-like transition dipoles localized at each molecule. Because of the H-aggregate geometry (Fig. 5f), the dipole-allowed state will be located at an energy higher than that of a monomer. In contrast, the unperturbed CT states may exhibit a different dependence on n. Assuming that CT occurs between two neighboring molecules and thus $\vec{\mu}_{A^+B^{-o}}$ is parallel to the stacking axis (***a*** axis for bulk crystals), the dipole allowed CT state of the n-mer will be a head-to-tail linear combination of constituent individual CT dipoles and have lower energy with increasing n because of the J-type dipole arrangements. These two trends suggest that the degree of the FE-

CT mixing becomes smaller because of the increasing energy difference between the two unperturbed states with increasing n. In other words, the CT dipole becomes more parallel to the LDS dipole, as depicted in Supplementary Figure 10a. This reasoning is consistent with the observed reorientation of CT dipoles in Fig. 5d. The other case may occur when the mixing coefficient in Equation S5 becomes larger, or the unperturbed FE dipole outweighs the unperturbed CT dipole with increasing thickness. As shown in Supplementary Figure 10b, the mixed CT dipole will eventually reorient and become more aligned with the LDS dipole.

Supplementary References

1. Möbus, M.; Karl, N.; Kobayashi, T. *J. Cryst. Growth* **1992,** 116, (3-4), 495-504.
2. Bulović, V.; Burrows, P.; Forrest, S.; Cronin, J.; Thompson, M. *Chem. Phys.* **1996,** 210, (1-2), 1-12.
3. Hoffmann, M.; Schmidt, K.; Fritz, T.; Hasche, T.; Agranovich, V. M.; Leo, K. *Chem. Phys.* **2000,** 258, (1), 73-96.
4. Hoffmann, M. Frenkel and Charge-Transfer Excitons in Quasi-One-Dimensional Molecular Crystals with Strong Intermolecular Orbital Overlap. Ph.D. Thesis, Technical University of Dresden, Dresden, Germany, 2000.
5. Hoffmann, M.; Soos, Z. G. *Phys. Rev. B* **2002,** 66, (2), 024305.
6. Yamagata, H.; Norton, J.; Hontz, E.; Olivier, Y.; Beljonne, D.; Bredas, J. L.; Silbey, R. J.; Spano, F. C. *J. Chem. Phys.* **2011,** 134, (20), 204703.
7. Koo, S.; Park, I.; Watanabe, K.; Taniguchi, T.; Shim, J. H.; Ryu, S. *Nano Lett.* **2021,** 21, (15), 6600-6608.
8. Harcourt, R. D.; Scholes, G. D.; Ghiggino, K. P. *J. Chem. Phys.* **1994,** 101, (12), 10521-10525.
9. Yamagata, H.; Pochas, C. M.; Spano, F. C. *J. Phys. Chem. B* **2012,** 116, (49), 14494-14503.
